# Editing of Chloroplast *rps14* by PPR Editing Factor EMB2261 Is Essential for *Arabidopsis* Development

**DOI:** 10.3389/fpls.2018.00841

**Published:** 2018-06-20

**Authors:** Yueming K. Sun, Bernard Gutmann, Aaron Yap, Peter Kindgren, Ian Small

**Affiliations:** Australian Research Council Centre of Excellence in Plant Energy Biology, School of Molecular Sciences, The University of Western Australia, Crawley, WA, Australia

**Keywords:** PPR, RNA editing, editing specificity, evolution, chloroplast, seed development, *Arabidopsis*

## Abstract

RNA editing in plastids is known to be required for embryogenesis, but no single editing event had been shown to be essential. We show that the *emb2261-2* mutation is lethal through a failure to express an editing factor that specifically recognizes the *rps14-2* site. EMB2261 was predicted to bind the *cis*-element upstream of the *rps14-2* site and genetic complementation with promoters of different strength followed by RNA-seq analysis was conducted to test the correlation between *rps14-2* editing and *EMB2261* expression. *Rps14-2* is the only editing event in *Arabidopsis* chloroplasts that correlates with *EMB2261* expression. Sequence divergence between the *cis*-element and the EMB2261 protein sequence in plants where *rps14-2* editing is not required adds support to the association between them. We conclude that EMB2261 is the specificity factor for *rps14-2* editing. This editing event converts P51 in Rps14 to L51, which is conserved among species lacking RNA editing, implying the importance of the editing event to Rps14 function. Rps14 is an essential ribosomal subunit for plastid translation, which, in turn, is essential for *Arabidopsis* embryogenesis.

## Introduction

RNA editing is a crucial process in plant organellar gene expression. In flowering plants, it involves cytidine (C) to uridine (U) deamination (Takenaka et al., 2013b). In *Arabidopsis thaliana*, over 600 C-to-U editing events have been detected in mitochondria and 44 C-to-U editing events have been detected in chloroplasts (Giege and Brennicke, 1999; Chateigner-Boutin and Small, 2007; Bentolila et al., 2013; Ruwe et al., 2013). RNA editing in plant organelles is facilitated by organelle-targeted pentatricopeptide repeat (PPR) editing factors (Barkan and Small, 2014). They contain multiple tandem helix-loop-helix PPR motifs that specifically bind to the RNA sequence just 5′ to the edited nucleotide in a one-motif to one-base manner, acting as site recognition factors (Barkan and Small, 2014). Amino acids at two positions in each PPR motif specifically recognize one of the four RNA bases, denoted as the PPR-RNA recognition code (Barkan et al., 2012; Takenaka et al., 2013a; Yagi et al., 2013a). This recognition appears to involve hydrogen bonding to the aligned RNA base (Shen et al., 2016). At the C-terminus of PPR proteins, there is a deaminase-like domain that is hypothesized to be part of a larger editosome (Sun et al., 2016). *Arabidopsis thaliana* encodes 216 potential PPR editing factors, forming one of its largest protein families (Cheng et al., 2016). Nineteen PPR editing factors have been identified accounting for 30 out of the 34 major editing sites in *Arabidopsis* chloroplasts (Kotera et al., 2005; Okuda et al., 2007, 2009; Chateigner-Boutin et al., 2008; Cai et al., 2009; Hammani et al., 2009; Robbins et al., 2009; Zhou et al., 2009; Hayes et al., 2013; Yagi et al., 2013b; Wagoner et al., 2015; Yap et al., 2015). Editing factors for the following four sites remained unidentified prior to this work: *ndhB-3* (96579), *ndhB-1* (97016), *petL* (65716), and *rps14-2* (37092).

Chloroplast biogenesis is essential to seed development. Mutations in genes involved in chloroplast gene expression such as those encoding ribosomal units (Tsugeki et al., 1996; Meinke et al., 2008) or splicing factors (Asakura and Barkan, 2006; Aryamanesh et al., 2017), can lead to premature arrest of embryogenesis during the globular to heart transition. RNA editing as an important post-transcriptional processing step in organelles is also known to be essential for seed development. For example, mutations in genes encoding DYW2 and NUWA required for RNA editing in both mitochondria and chloroplasts are embryo-lethal (Andres-Colas et al., 2017; Guillaumot et al., 2017). Some site-specific PPR editing factors targeted to mitochondria, such as EMP9 (Yang et al., 2017) and DEK36 (Wang et al., 2017) are also essential during seed development. However, mutants of the 19 site-specific PPR editing factors in *Arabidopsis* chloroplasts described prior to this work are all viable, showing a variety of phenotypes at later developmental stages, including decreased chloroplast NDH complex activity (Kotera et al., 2005; Okuda et al., 2007, 2009; Cai et al., 2009), changes in leaf pigmentation (Chateigner-Boutin et al., 2008; Cai et al., 2009; Zhou et al., 2009; Wagoner et al., 2015; Yap et al., 2015) and aberrant leaf shapes (Hayes et al., 2013). Only one embryo-lethal mutation (*emb2261*) that affects a potential site-specific PPR editing factor in *Arabidopsis* chloroplasts has been described (Cushing et al., 2005). The *emb2261* mutant stalls at the heart stage during seed development. We sought to characterize the function of EMB2261 and its potential target site(s).

One approach to study embryo-lethal mutants is to perform partial complementation. The use of the seed-specific *ABI3* promoter to drive the gene of interest for partial complementation has been successful in studying the embryo-lethal mutants *emb506* (Despres et al., 2001), *emb2394* and *emb2654* (Aryamanesh et al., 2017), among which *emb2654* is a chloroplast PPR splicing factor mutant. We therefore hypothesized that *ABI3*-promoter-driven *EMB2261* constructs could partially complement the *emb2261* mutant such that it could complete seed development. *EMB2261* expression would then fade away as the *ABI3* promoter loses its activity, leaving only the *emb2261* mutant background from the seedling stage onward. Therefore, the partial complementation method would provide an opportunity to obtain enough plant tissue to examine RNA editing in the *emb2261* mutant background.

During the writing up of this work, an independent manuscript reported that ECD1 (synonymous with EMB2261) is required for editing of the *rps14-2* site in *Arabidopsis* and is required for early chloroplast development (Jiang et al., 2018). We confirm this conclusion with a different genetic approach and a different mutant allele of the *EMB2261* gene, and expand these findings by considering the specificity of the EMB2261/*rps14-2* interaction and the evolutionary history of the pair.

## Materials and Methods

### Prediction Method

The alignment of an editing factor and a site was scored by calculating the sum of the log-likelihood ratios at each position in the alignment (Yap et al., 2015). The log-likelihood ratios were derived from observed frequencies of association between amino acid combinations at the fifth and last position and the four RNA nucleotides (Supplementary Table S1). Histograms were generated using matplotlib v1.5.3^1^. Heat maps were generated using v0.8.1^2^.

### Cloning of Plant Transformation Constructs

The *EMB2261* gene fragment was amplified from Col-0 genomic DNA with the *attB* recombination sites introduced using PrimeSTAR polymerase (Clontech^3^). The *EMB2261* PCR product was purified by QIAquick PCR purification kit (Qiagen^4^), cloned into the donor vector pDONR207 using Gateway BP Clonase (Invitrogen^5^). The *EMB2261* gene fragment was then cloned from the entry vector pDONR207 to the plant expression vector pH7WG containing the *ABI3* promoter (Aryamanesh et al., 2017) (*ABI3*:*EMB2261*), or pGWB2 (EMBL) containing the 35S promoter (*35S*:*EMB2261*), using Gateway LR Clonase (Invitrogen, see footnote 5). Cloning reactions were transformed into *E. coli* competent cells (DH5α). Positive clones for each construct were confirmed by Sanger sequencing. The verified plant expression constructs were transformed into *Agrobacterium tumefaciens* competent cells (GV3101).

### Plant Growth, Transformation, and Selection

*Arabidopsis* seeds were surface sterilized with 70% ethanol supplemented with 0.05% Triton-X100 for 5 min and washed with 100% ethanol before being dried in the fume hood. Sterilized seeds were sowed on plates (half-strength MS medium and 0.8% agar), stratified at 4°C in the dark for 3 days, germinated and grown under long-day conditions (16 h light/8 h dark cycle, approximately 120 μmol photons m^-2^ s^-1^). Heterozygous plants of *emb2261-2* (SALK_024975) were selected by genotyping using the primer pair SALK_024975_RP (CTTTCTCGAGTGCATTCAAGG) and LBb1.3 (ATTTTGCCGATTTCGGAAC) for T-DNA insertion, and with the primer pair SALK_024975_RP (CTTTCTCGAGTGCATTCAAGG) and SALK_024975_LP (TATATTTGGTGAGCATTCGGG) for genomic DNA. Plants were transformed by floral dip (Clough and Bent, 1998). Seeds harvested from the dipped plants were germinated and selected on Hygromycin B (25 μg/ml). Transformants were genotyped for homozygosity of the T-DNA insertion in the *EMB2261* gene with the same set of primers listed above, except that the reverse primer SALK_024975_LP2 (GTGTATCTAAATCTCAAAGTCACC) annealing to the 3′UTR of the native *EMB2261* gene was used to distinguish between the native *EMB2261* gene and the *EMB2261* transgenes.

### RNA Analysis

Total RNA was isolated using the PureZOL reagent (Bio-Rad^6^) and treated with TURBO DNase (Ambion^7^) according to the manufacturer’s instructions. Completion of DNase treatment was verified by PCR targeting chloroplast genomic DNA. Complementary DNA (cDNA) was synthesized using random primers and SuperScript III reverse transcriptase (Invitrogen, see footnote 5) according to the manufacturer’s instructions.

The primer pair targeting the *rps14-2* editing site were TCGCTAAGTGAGAAATGGAAAA (forward) and CGTCGATGAAGACGTGTAGG (reverse). The PCR cycling conditions were 40 cycles of 10 s at 98°C, 15 s at 58°C, and 4 s at 72°C, using PrimeSTAR polymerase (Clontech, see footnote 3). Poisoned primer extension (PPE) was carried out as described by Chateigner-Boutin and Small (2007) with a nucleotide mix containing dideoxythymidine (ddT). The fluorescein-labeled primer used for PPE was 6′FAM-AAATGGAAAATTCATGGAAAATTACAAT.

The quantitative PCR (qPCR) primer pair targeting the *EMB2261* gene were CGTACGTTTCTTGGAGCTTGCAG (forward) and TTCCCATTTCCCTGCACAAGCG (reverse). qPCR was performed using the Quantinova mix (Qiagen, see footnote 4) according to manufacturer’s instructions on a Lightcycler 480 machine (Roche Molecular Diagnostics^8^). *EMB2261* gene expression was normalized to expression of the reference gene *CACS* by the formula: (1 + E_EMB2261_)ˆ(35-Cq_EMB2261_)/(1 + E_CACS_)ˆ(35-Cq_CACS_).

### RNA-Seq and Data Analysis

RNA-seq libraries were prepared using TruSeq Stranded Total RNA LT Kit with Ribo-zero plant (Illumina^9^), quantified using KAPA Library Quantification Kit for Illumina platforms (Kapa Biosystems, KK4854^10^), and pooled in an equimolar ratio. Single-end sequencing was performed with a read length of 61 bases on an Illumina HiSeq 1500 sequencer. The sequence datasets are available in the NCBI Sequence Read Archive (SRA) repository (accession SRP141099).

The data in fastq format was trimmed using Trimmomatic v0.33 (Bolger et al., 2014) to remove the adapter sequence (ILLUMINACLIP:TruSeq_index.fasta:2:30:3, TruSeq_index =CAAGCAGAAGACGGCATACGAGAT), bases with Phred Quality score < 20 (LEADING:15 TRAILING:15 SLIDINGWINDOW:4:20) and reads shorter than 30 bases (MINLEN:30). All the reads were then reverse complemented using seqtk v1.2-r102-dirty^11^ before mapping with STAR v020201 (Dobin et al., 2013). The index was built upon the TAIR10 genome and annotation (Lamesch et al., 2012) with the following modifications: (1) The concatenated *rps12* gene (Aryamanesh et al., 2017), namely rps12A-intron1a-intron1b-rps12B with 60 bp extra at each end, was appended to the end of the chloroplast genome (ChrC:154479–156997); (2) The coordinates of concatenated *rps12* intron 1 and intron 2 are set as 154653–156142 and 156375–156911, respectively; (3) The coordinates of *ycf3* intron 1 is shifted one nucleotide downstream to 43753–44466. The reads were aligned with the following parameters: –outFilterMismatchNmax 4, –outSAMprimaryFlag AllBestScore, –alignIntronMax 1 and –outSAMtype BAM SortedByCoordinate. The alignments of the highest scores were selected (view –bF 0x100) and indexed with samtools v1.3.1 (Li et al., 2009).

For the editing analysis, pileup files were generated using pysamstats v1.0.1^12^ using the parameters –d, -D 100,000,000 and –type variation_strand and filtered as followed: (1) the nucleotide is encoded as C on the examined strand of the *Arabidopsis* chloroplast genome; and (2) the number of putatively edited reads (containing a T instead of a C at the site) was greater than 10, and the proportion of putatively edited reads was greater than 1%. Potential editing events induced by *EMB2261* expression were looked for as follows: (1) the editing event was detected in all three samples of *35S*:*EMB2261*; (2) the ‘edited’ reads were not simply mis-aligned, especially where ‘edited’ position is in the sequence context (T)_n_C(T)_n_ on the forward strand or (A)_n_G(A)_n_ on the reverse strand; and (3) the proportion of edited reads followed the pattern of *ABI3*:*EMB2261* = < Col-0 < *35S*:*EMB2261*.

For the splicing analysis, the splicing function of the ChloroSeq package (Castandet et al., 2016) was used with the same adjustments as described above made to the annotation files. For the gene expression analysis, the count for each gene was obtained using featureCounts v1.5.3 (Liao et al., 2014). Ribosomal RNA genes, tRNA genes, the non-concatenated *rps12* exons and one copy of the inverted repeat region were excluded. The RPKM (Reads Per Kilobase of transcript per Million mapped reads) values were calculated using the formula $\frac{Ri}{TiL}$, where C is a constant of 10^9^, *Ri* is the number of reads per gene of interest, *Ti* is the total number of reads mapped to the gene set of interest, and *L* is the length of the gene of interest.

### Evolution Analyses

The *rps14* sequences were extracted from the chloroplast genomes deposited in NCBI Genbank (Supplementary Table S2). The orthologs of EMB2261 were identified in the plantPPR database (Cheng et al., 2016) by BLAST (v2.2.29+) search (Camacho et al., 2009). The PPR protein sequences from species where no genome sequence is available were retrieved by BLAST from NCBI Genbank^13^. We selected a single best sequence for each species when multiple matches to *Arabidopsis* EMB2261 were reported (Supplementary Table S2).

The alignment was carried out using the MAFFT v.7 online server (Katoh et al., 2017) and trimmed with trimAl using default parameters (Capella-Gutierrez et al., 2009). A phylogenetic tree was inferred by maximum likelihood with *Amborella trichopoda* EMB2261 as an outgroup, using IQ-Tree-omp v1.5.5 (Nguyen et al., 2015). The optimal evolutionary model (JTT + F + I + G4) was selected by ModelFinder (Kalyaanamoorthy et al., 2017). Branching support was estimated from 100 standard non-parametric bootstrap replicates.

The consensus sequence logos of the PPR binding sites and PPR motifs were generated by Weblogo^14^. To compare the conservation of the 12th PPR motif in different families of monocots, alignments were generated using the MAFFT v.7 online server and then submitted to the EMBOSS plot conservation tool^15^.

## Results

### PPR Editing Factor EMB2261 Is Predicted to Edit *rps14-2* in *Arabidopsis* Chloroplasts

**FIGURE 1:**
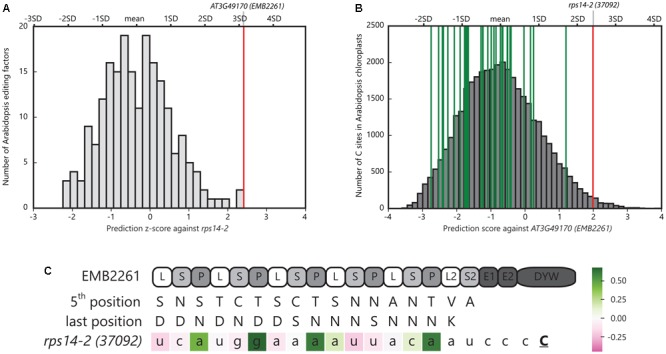
Pentatricopeptide repeat (PPR) editing factor EMB2261 is predicted to edit *rps14-2* in *Arabidopsis* chloroplasts. **(A)** Histogram of *z*-scores of the *Arabidopsis rps14-2* editing site aligned against all putative *Arabidopsis* PPR editing factors (*n* = 204). The *z*-scores were calculated as the number of standard deviations the *rps14-2* alignment score was from the mean prediction score of all possible alignments in the chloroplast genome for that editing factor. The red line indicates the highest scoring alignment against *rps14-2*, representing AT3G49170 (EMB2261). **(B)** Distribution of alignment scores of potential editing sites (YC) across the *Arabidopsis* chloroplast genome (*n* = 37,888) against AT3G49170 (EMB2261). The red line indicates the *rps14-2* alignment; green lines indicate the alignments to the other 33 major chloroplast editing sites. **(C)** Alignment of EMB2261 motifs with the *rps14-2* editing site in *Arabidopsis* chloroplasts. **C** indicates the edited cytidine. The nucleotides are colored according to the alignment scores (Supplementary Table S1). The P1, P2, L1, and S1 motifs are labeled as P, L, and S motifs for simplicity.

EMB2261 is located in chloroplasts in *Arabidopsis* (Tanz et al., 2013). The *rps14-2* site in *Arabidopsis* chloroplasts is predicted to be edited by EMB2261 (encoded by *AT3G49170*) according to the PPR-RNA code (Barkan et al., 2012). As shown in **Figure 1A**, EMB2261 scores the highest among all *Arabidopsis* editing factors against the *rps14-2* site. Moreover, as shown in **Figure 1B**, *rps14-2* scores the highest among all major editing sites in *Arabidopsis* chloroplasts against EMB2261. EMB2261 motifs align with the *rps14-2* editing site (**Figure 1C**).

### Editing of *rps14-2* Correlates With *EMB2261* Expression and Is Essential for *Arabidopsis* Seed Development

**FIGURE 2:**
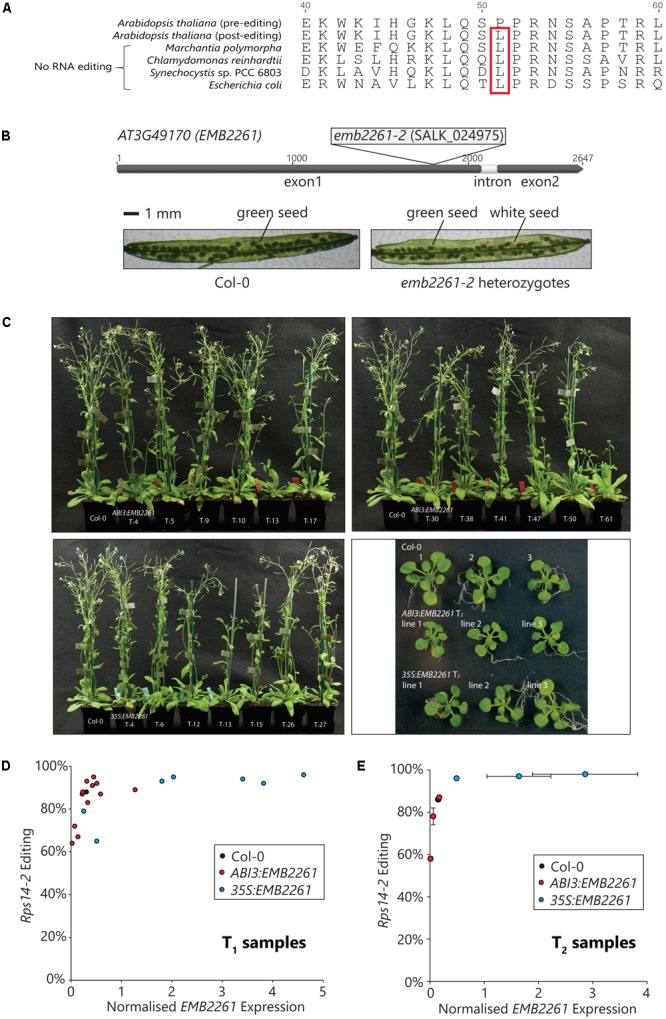
Editing of *rps14-2* correlates with *EMB2261* expression and is essential for *Arabidopsis* seed development. **(A)** Conservation of the edited *rps14-2* codon in species lacking RNA editing. *Rps14-2* editing converts the second position of the 51st codon in Rps14 from C to U, changing the amino acid P51 to L51. L51 is encoded in the *rps14* genomic sequence from species lacking RNA editing, such as *Marchantia polymorpha, Chlamydomonas reinhardtii, Synechocystis* sp. PCC 6803, and *Escherichia coli*. **(B)** Embryo-lethality of the *emb2261-2* mutation. The SALK_024975 mutant line carries a T-DNA insertion in the first exon of the *EMB2261* (*AT3G49170*) gene. Seeds of heterozygous *EMB2261-2*/*emb2261-2* plants segregate into two phenotypes, green and white, at a ratio of 3 to 1. No homozygous mutant plants can be recovered. **(C)** Complementation of the *emb2261-2* mutation with *ABI3*:*EMB2261* and *35S*:*EMB2261*. Mature plants of primary transformants (T_1_) expressing *ABI3*:*EMB2261* or *35S*:*EMB2261* in a homozygous *emb2261-2* mutant background, in comparison with wild type Col-0; and seedlings (18-day-old) of three independent transgenic lines (T_2_) expressing *ABI3*:*EMB2261* or *35S*:*EMB2261* in a homozygous *emb2261-2* mutant background, in comparison with wild type Col-0. *ABI3*:*EMB2261* lines 1, 2, and 3 were derived from the primary transformants T_1_13, T_1_41, and T_1_61, respectively. *35S*:*EMB2261* lines 1, 2, and 3 were derived from the primary transformants T_1_12, T_1_15, and T_1_26, respectively. **(D)** Correlation between *rps14-2* editing and *EMB2261* expression in T_1_ transgenic plants. From the flowering tissue of each T_1_ plant shown in **(C)**, *rps14-2* editing was quantified by poisoned primer extension (PPE), and *EMB2261* expression was quantified by RT-qPCR normalized to the expression of the reference gene *CACS* (*AT5G46630*). *Rps14-2* editing is plotted against *EMB2261* expression. Each data point represents an individual transgenic line. **(E)** Correlation between *rps14-2* editing and *EMB2261* expression in T_2_ transgenic plants. From the whole seedling (T_2_) tissue shown in (c), *rps14-2* editing was quantified by PPE, and *EMB2261* expression was quantified by RT-qPCR normalized to the expression of the reference gene *CACS* (*AT5G46630*). *Rps14-2* editing is plotted against *EMB2261* expression. Each data point represents an individual transgenic line. Both horizontal and vertical error bars show SE, *n* = 3.

*Rps14-2* editing changes the 51st codon of the *rps14* transcript from CCA, encoding proline (P), to CUA, encoding leucine (L). As shown in **Figure 2A**, the genomic *rps14* sequences from *E. coli*, as well as examples of other species that lack RNA editing, encode L51 instead of P51, implying that L51 is important to Rps14 function. According to the structure reported for the chloroplast 70S ribosome (Bieri et al., 2017), L51 is in a loop that is in close contact with the ribosomal RNA. Proline is a poor substitute for flexible amino acids (e.g., leucine) in protein structures, and the L51P mutation is likely to change Rps14 structure and function.

Consistent with the previous characterization of an *emb2261* mutation (Cushing et al., 2005), the T-DNA insertion in the line SALK_024975 (*emb2261-2*) is embryo-lethal. As shown in **Figure 2B**, the T-DNA insertion was mapped directly after the nucleotide 1809 of the *EMB2261* gene, within the region encoding the L2 motif. Siliques of the heterozygous *EMB2261-2*/*emb2261-2* plants contain three quarter green seeds and one quarter white seeds. No homozygous plants (*emb2261-2*/*emb2261-2*) could be recovered.

To investigate the relationship between EMB2261 and *rps14-2* editing, *emb2261-2* heterozygotes were transformed with either the *ABI3*:*EMB2261* or the *35S*:*EMB2261* construct. It was expected that the *ABI3*:*EMB2261* primary transformants would segregate into two phenotypes. Plants that carry the transgene in wild type or heterozygous mutant background would look like wild type, and plants that carry the transgene in homozygous mutant background would show a strong chloroplast-deficient phenotype as the *ABI3* promoter activity fades away. However, all plants resembled the wild type (**Figure 2C**). Subsequent genotyping revealed that around 1/5 (12 out of 62) seedlings carry the *ABI3*:*EMB2261* transgene in a homozygous mutant background. These results indicate that both *ABI3*:*EMB2261* and *35S*:*EMB2261* complement the embryo-lethal phenotype of *emb2261-2*, i.e., that the residual *ABI3* promoter activity beyond the seed stage drives sufficient expression of *EMB2261* for normal embryo and seedling development. Seven *35S*:*EMB2261* lines in the homozygous mutant background were isolated.

We then sought to check whether there were any subtle *rps14-2* editing defects in the *ABI3*:*EMB2261* lines. All the primary transformants were screened for *EMB2261* gene expression and *rps14-2* editing, in comparison with wild type Col-0. Flower tissues were harvested from the primary transformants and Col-0 for RNA analyses (**Figure 2C**). *EMB2261* expression was quantified by qPCR (Supplementary Figure S1A), and *Rps14-2* editing was quantified by PPE (Supplementary Figure S1B). As shown in **Figure 2D**, the proportion of *rps14-2* editing was plotted against the normalized *EMB2261* expression value. *Rps14-2* editing correlates with *EMB2261* expression in the primary transformants. In general, *ABI3*:*EMB2261* lines show decreased or comparable *EMB2261* expression level to wild type, correlating with lower or comparable *rps14-2* editing level. *35S*:*EMB2261* over-expression lines show increased *EMB2261* expression and almost 100% *rps14-2* editing.

To confirm the correlation between *rps14-2* editing and *EMB2261* expression, the analysis was repeated in the second generation (T_2_) of the transgenic lines. Three *ABI3*:*EMB2261* lines that showed lower than wild-type levels of *EMB2261* expression and three *35S*:*EMB2261* lines that showed higher than wild-type levels of *EMB2261* expression were selected, and whole 18-day-old seedlings were harvested for RNA analyses (**Figure 2C**). In **Figure 2E**, the proportion of *rps14-2* editing (Supplementary Figure S1D) is plotted against the normalized *EMB2261* expression value (Supplementary Figure S1C). The correlation between *rps14-2* editing and *EMB2261* gene expression is maintained in the T_2_ generation. Taking the above results together, we conclude that *EMB2261* is an editing specificity factor for the *rps14-2* site.

### *Rps14-2* Is the Only Chloroplast Editing Event That Positively Correlates With *EMB2261* Expression

If EMB2261 edits other sites, their editing level should also correlate with *EMB2261* gene expression, following a similar pattern to editing of the *rps14-2* site. We quantified the editing level at all known chloroplast editing sites in *ABI3*:*EMB2261*, wild type Col-0, and *35S*:*EMB2261* by RNA-seq.

**FIGURE 3:**
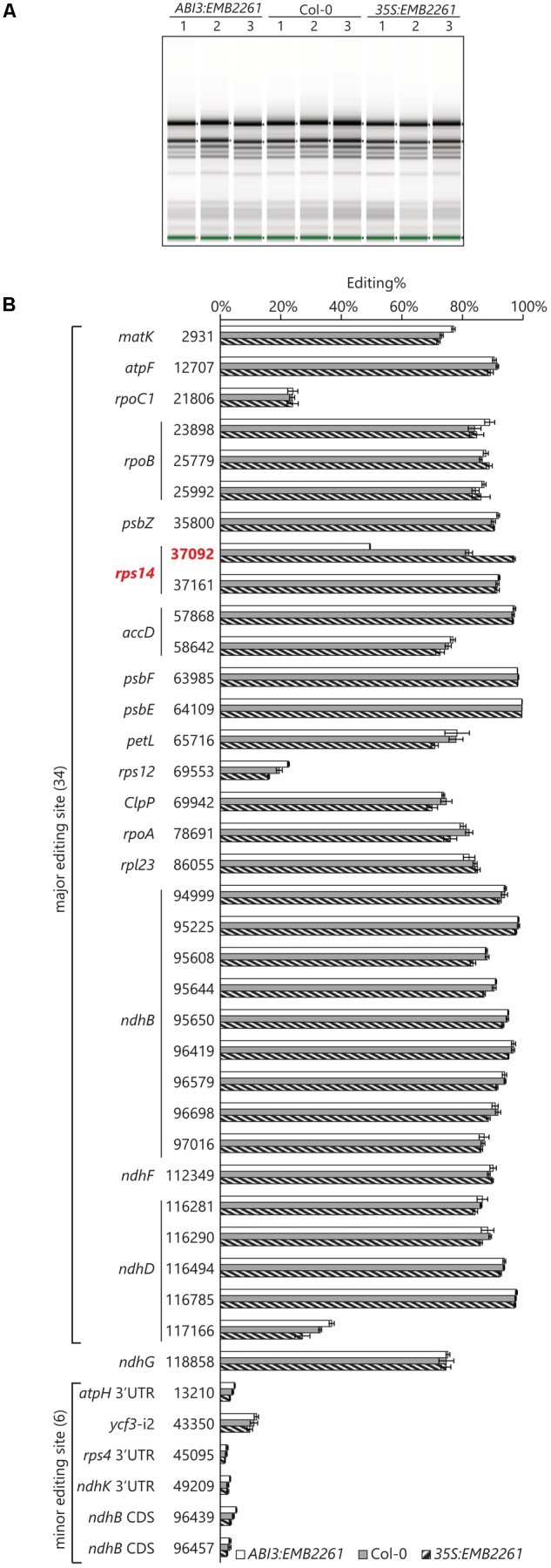
*Rps14-2* is the only known chloroplast editing event that positively correlates with *EMB2261* expression. **(A)** Screentape image of RNA samples extracted from *ABI3*:*EMB2261* line 1 and *35S*:*EMB2261* line 2, in comparison with wild type Col-0. Each lane represents one RNA sample extracted from a single 18-day-old seedling (T_2_). **(B)** RNA editing quantified at known chloroplast editing sites in *ABI3*:*EMB2261* and *35S*:*EMB2261*, in comparison with wild type Col-0. Error bars show SE, *n* = 3.

The transgenic line showing the lowest *EMB2261* expression level was chosen for *ABI3*:*EMB2261*, and the transgenic line showing the highest *EMB2261* expression level was chosen for *35S*:*EMB2261*. A separate batch of 18-day-old T_2_ seedlings was obtained, including three seedlings as three biological replicates for each of *ABI3*:*EMB2261, 35S*:*EMB2261*, and wild type Col-0. Prior to RNA-seq library preparation, RNA quality was checked with an Agilent Screentape. There is no difference between the genotypes in terms of ribosomal RNA accumulation (**Figure 3A**), indicating that ribosome assembly in *ABI3*:*EMB2261* is not greatly different from wild type, despite reduced editing of *rps14-2*. This observation is consistent with the lack of visible growth phenotypes in *ABI3*:*EMB2261*.

About 33 million reads were obtained for each sample, roughly 60% of which aligned to the *Arabidopsis* chloroplast genome. Editing at known chloroplast editing sites were quantified as (number of T reads)/(number of T reads + number of C reads)%. Only *rps14-2* editing positively correlates with *EMB2261* expression (**Figure 3B**). Some major editing events [e.g., *rps12(69553)*] may correlate negatively with *EMB2261* expression, although to a lesser extent than the positive correlation with *rps14-2*. In addition, six out of seven previously reported minor editing events (Bentolila et al., 2013; Ruwe et al., 2013) were detected in all nine samples, none of which correlate with *EMB2261* expression (**Figure 3B**). After examining all possible editing events across the chloroplast transcriptome, not limited to the known chloroplast editing sites, we found no novel editing events that were induced by *EMB2261* overexpression (Supplementary Table S3). Notably, potential editing sites that match EMB2261 motifs better than *rps14-2* were confirmed not to be edited (Supplementary Figure S2). Lack of induced editing events upon overexpression indicates that EMB2261 is a highly specific editing factor in *Arabidopsis* chloroplasts. In addition, we also quantified chloroplast splicing efficiency (Supplementary Figure S3) and gene expression (Supplementary Figure S4) based on the RNA-seq data. We found no transcripts or processing events besides *rps14-2* editing that strongly depended on *EMB2261* expression.

### Both EMB2261 and the Recognition Sequence Are Subject to Divergence in Species Where *rps14-2* Editing Is no Longer Needed

**FIGURE 4:**
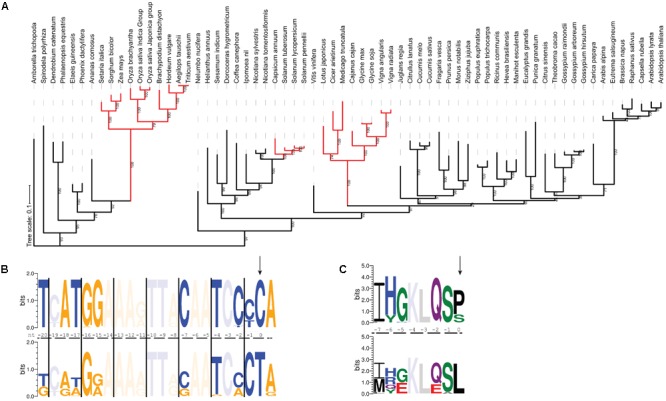
The RNA recognition sequence diverges in species where *rps14-2* editing is no longer needed. **(A)** Maximum likelihood tree of EMB2261 orthologs from 66 species (Supplementary Table S2). Branches where *rps14-2* editing is no longer needed are highlighted in red. **(B)** Conservation of *cis*-elements immediately upstream of the *rps14-2* editing site. The top panel shows the sequence logo generated from *rps14* sequences from 43 plant species where the *rps14-2* site requires editing. The bottom panel shows the sequence logo generated from *rps14* sequences from 23 plant species where the equivalent position is a T in the chloroplast genome. The *rps14-2* editing site is highlighted by the arrow. Purines are indicated in orange and pyrimidines in blue. Positions where there are no new nucleotides introduced in either group are faded. **(C)** Conservation of the *cis*-elements shown in **(B)** translated to amino acids. The *rps14-2* editing site is highlighted by the arrow. Positions where there are no new amino acids introduced in either group are faded.

The *rps14-2* site has been lost three times during evolution in Solanaceae, Fabaceae and Poaceae, respectively (**Figure 4A**), where T instead of C is present in the chloroplast genomes. In these species, the *cis*-element immediately upstream of the *rps14-2* editing site is less conserved than when editing is required. As shown in **Figure 4B**, positions -7, -15, -17, -18, and -20 show variation from the consensus once *rps14-2* no longer needs to be edited. This nucleotide variation introduces variation in the corresponding amino acid sequence (**Figure 4C**), apparently without affecting Rps14 function.

Putative *EMB2261* orthologs are still present in all three families. The C-terminal domains including the DYW domain are conserved (Supplementary Figure S5A). Despite the loss of *rp14-2* editing site in Solanoideae (*Solanum* and *Capsicum*), a subfamily of Solanaceae, the protein sequence of putative *EMB2261* orthologs remain conserved (Supplementary Table S4). EMB2261 protein sequences in Fabaceae species show slightly more variation compared to those in other dicot species (Supplementary Table S4). EMB2261 proteins in Poaceae species show more dramatic sequence variation compared to those in other monocot species, especially in one truncated P1 motif (Supplementary Figures S5B,C). Thus, the absence of *rps14-2* editing can be associated with sequence divergence in EMB2261.

## Discussion

With a different genetic approach based on a different mutant allele of the *EMB2261* gene, we confirm the conclusion of Jiang et al. (2018), claiming that EMB2261/ECD1 is the editing specificity factor of *rps14-2* in *Arabidopsis* chloroplasts and is essential for *Arabidopsis* development. Here, we discuss the implication of this finding from four aspects: (1) editing factor mutants as surrogate mutants of the corresponding genes; (2) PPR specificity factors as limiting factors in RNA editing; (3) editing specificity determinants beyond the PPR-RNA recognition code; and (4) co-evolutionary scenarios involving PPR RNA editing factors and their targets.

### Lethality of Editing Defects

RNA editing events in plant organelles mainly occur in the coding region of genes and alter the corresponding protein sequences. In mutants lacking one of the site-specificity PPR editing factors, the editing site(s) targeted by the factor remain(s) completely unedited, often leading to functional defects of the gene products encoded by the affected transcripts. Therefore, editing factor mutants appear as surrogate mutants of the corresponding organellar genes. As most chloroplast genes encode subunits of the photosynthetic machinery, it is thus not surprising that most chloroplast editing mutants show photosynthetic defects. Photosynthesis is not essential for embryogenesis, but plastid translation is essential (in most plants) due to the requirement for the plastid-encoded AccD (acetyl-coA carboxylase D) gene product, the loss of which results in embryonic lethality (Bryant et al., 2011). Thus, loss of RNA editing of plastid-encoded essential components of the translation machinery could conceivably cause embryonic lethality. Rps14 is known to be an essential ribosomal subunit in tobacco chloroplasts (Ahlert et al., 2003) and in *E. coli* (Shoji et al., 2011). We contend that this is the most likely explanation for the *emb2261* phenotype: EMB2261 is required for editing at the *rps14-2* site, which in turn is required for synthesis of functional Rps14, which in turn is required for plastid translation.

### EMB2261 Is a Limiting Factor for *rps14-2* Editing

Partial complementation of *emb2261-2* by *ABI3*:*EMB2261* did not work as we expected. Comparing the expression profiles (Schmid et al., 2005) of *ABI3, EMB2261*, and the other successfully partially complemented *EMB* genes (Despres et al., 2001; Aryamanesh et al., 2017), we found that the expression level of the other *EMB* genes are 10–100 times higher than *ABI3* beyond the seed stage, whereas the expression level of *EMB2261* is of the same order of magnitude as *ABI3* (Supplementary Figure S6). Therefore, the residual *EMB2261* expression driven by *ABI3* promoter beyond the seed stage is likely to have been sufficient to complement the *emb2261* mutant phenotype. Since *ABI3* is considered to be a seed-specific gene (Cushing et al., 2005), the required *EMB2261* expression level must be very low. That *rps14-2* editing is increased upon *EMB2261* overexpression indicates that it is not saturated and that a limiting factor is the expression of *EMB2261*.

Unlike EMB2261, some PPR specificity factors lack the essential C-terminal editing domains that have to be supplied *in trans* (Andres-Colas et al., 2017; Diaz et al., 2017; Guillaumot et al., 2017). In these cases, the PPR specificity factor itself may not be the limiting factor for editing. For example, CRR4 lacks the DYW domain, which is complemented by the DYW1 protein *in trans* (Boussardon et al., 2012). The editing of *ndhD-1* by CRR4 is not correlated with *CRR4* expression (Kotera et al., 2005), but can be boosted by overexpression of a CRR4-DYW1 fusion (Boussardon et al., 2012).

### Editing Specificity

The specificity of PPR editing factors is predominantly determined by the interaction between the fifth and last position of a PPR motif and the aligned RNA base. The one-motif to one-base recognition code forms the basis of editing site prediction methods (Barkan et al., 2012; Takenaka et al., 2013a; Yagi et al., 2013a). However, if these are the only determinants of RNA editing specificity, it is surprising that there are not more editing events in chloroplasts than are observed. For example, there are hundreds of potential editing sites in *Arabidopsis* chloroplasts that match the EMB2261 PPR motifs equally well or better than *rps14-2* (Supplementary Figure S2), yet only one editing event was unambiguously detected in this work. The lack of off-target editing events implies that there are additional factors other than the canonical PPR-RNA code controlling the editing specificity of EMB2261.

Pentatricopeptide repeat motifs may contribute differently to RNA recognition. Although the predicted candidate sites shown in Supplementary Figure S2 score higher than *rps14-2* based on the PPR-RNA code, they show different distributions of mis-matches across the PPR-RNA alignments compared to *rps14-2*. It may be that EMB2261 motifs matching *rps14-2* play more important roles in target recognition, and the potential editing sites containing mismatches to these motifs are not likely to be recognized. There may also be non-canonical, yet sequence-specific, interactions between PPR motifs and target RNA that are not currently taken into account. In the case of EMB2261, the PPR motifs aligned to positions -16, -13, and -8 contain non-canonical combinations of amino acids, the selectivity of which is poorly understood due to lack of prior examples.

Not all predicted sites may be expressed or accessible to PPR editing factors *in planta*, where RNA forms secondary structures or is bound by other proteins. RNA secondary structure prevents PPR binding *in vitro* (Kindgren et al., 2015; Miranda et al., 2018), implying that it would inhibit RNA editing *in planta*. RNA structures and protein-RNA interactions can be partially modulated by RNA chaperones and helicases. For example, knock-down of chloroplast RNA helicase ISE2 leads to specific editing defects at 12 sites, including *rps14-2* (Bobik et al., 2017). Therefore, *rps14-2* editing by EMB2261 may also require ISE2, and lack of *rps14-2* editing in *ise2* null mutants may be one of the reasons that the mutation is lethal. There is also an association shown by RNA immunoprecipitation sequencing (RIP-seq) between ISE2 and *rps14* transcripts as well as other edited chloroplast transcripts. Taken together, these observations imply that the chloroplast RNA helicase ISE2 may be required to remodel RNA structures and/or protein-*rps14* interactions near the *rps14-2* site.

### Co-evolution Between Editing Factors and Editing Sites

Co-evolution between PPR editing factors and their target sites has been demonstrated in two scenarios. First, PPR editing factors and editing sites tend to be gained in parallel. For example, at the current limits of phylogenetic resolution, the mitochondrial PPR editing factor PPR_56 in *Physcomitrella patens* appeared simultaneously with its two editing sites on *nad3* and *nad4* (Beike et al., 2014). Second, PPR editing factors and editing sites tend to also be lost in parallel. For example, loss of the chloroplast editing factors CRR28 and RARE1 coincide with loss of their corresponding editing sites (Hein et al., 2016). Editing factors targeting multiple sites tend to be retained as long as a subset of their targeting sites remains (Hein et al., 2016). EMB2261 may have more than one target site in species other than *Arabidopsis*, which would explain the conservation of EMB2261 despite the loss of the *rps14-2* editing event. The patterns of sequence variation in the *cis*-element immediately upstream of *rps14-2* suggest that it is conserved in species that edit this site due to the requirement for RNA recognition rather than because of protein sequence conservation. When editing is no longer needed, the *cis*-element of the editing site is free to diverge to the extent that the corresponding amino acid changes can be functionally tolerated. Co-evolution of editing factors and their binding sites may be a powerful way of examining sequence recognition by PPR proteins, where a sufficient number of independent examples of editing loss and subsequent sequence divergence can be compared.

## Author Contributions

YS, BG, AY, PK, and IS designed the research and wrote the manuscript. YS performed the experiments. YS, BG, and IS collected, analyzed, and interpreted the data.

## Conflict of Interest Statement

The authors declare that the research was conducted in the absence of any commercial or financial relationships that could be construed as a potential conflict of interest.

## Footnotes

1 www.matplotlib.org

2 seaborn.pydata.org

3 www.clontech.com

4 www.qiagen.com

5 www.thermofisher.com/au/en/home/brands/invitrogen

6 www.bio-rad.com

7 www.thermofisher.com/au/en/home/brands/invitrogen/ambion

8 www.molecular.roche.com

9 www.illumina.com

10 www.kapabiosystems.com

11 github.com/lh3/seqtk

12 github.com/alimanfoo/pysamstats

13 http://www.ncbi.nlm.nih.gov/blast/Blast.cgi

14 http://weblogo.berkeley.edu

15 http://emboss.bioinformatics.nl/cgi-bin/emboss/plotcon
