# Supplementary material for: Editing of Chloroplast rps14 by PPR Editing Factor EMB2261 Is Essential for Arabidopsis Development
Source: Front Plant Sci. 2018 Jun 20;9:841. doi: 10.3389/fpls.2018.00841 (PMC6019781; doi:10.3389/fpls.2018.00841)
Supplement: Supplementary file 5 [file Image_1.PDF]

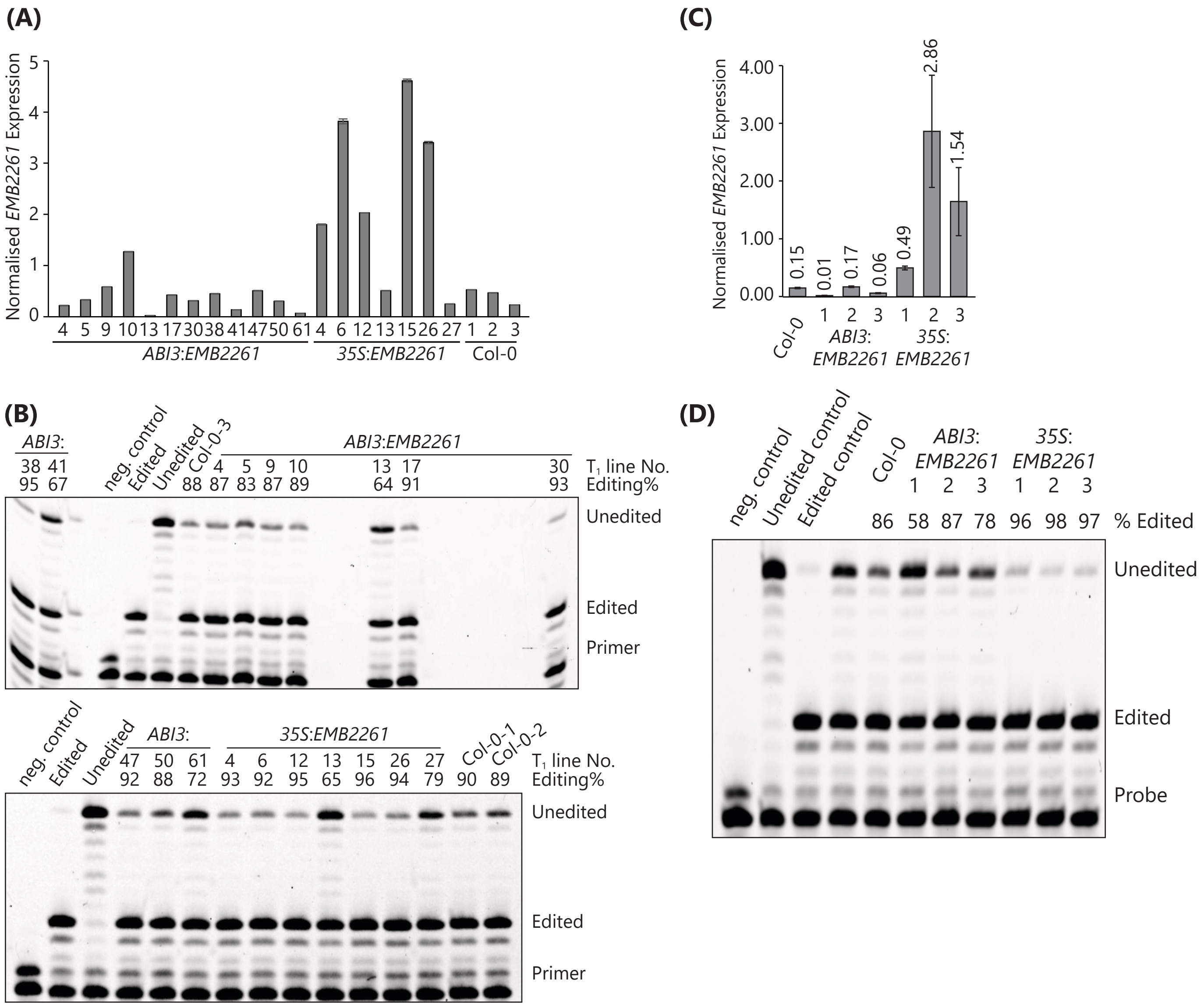

FIGURE S1. *EMB2261* gene expression and *rps14-2* editing quantification.

(A) Normalised *EMB2261* expression in T_1 transgenic plants.

(B) Editing of *rps14-2* in T_1 transgenic plants.

(C) Normalised *EMB2261* expression in T_2 transgenic plants. Error bars show SE, n=3.

(D) A representative PPE gel (one of three replicates) indicating *rps14-2* editing in T_2 transgenic plants.

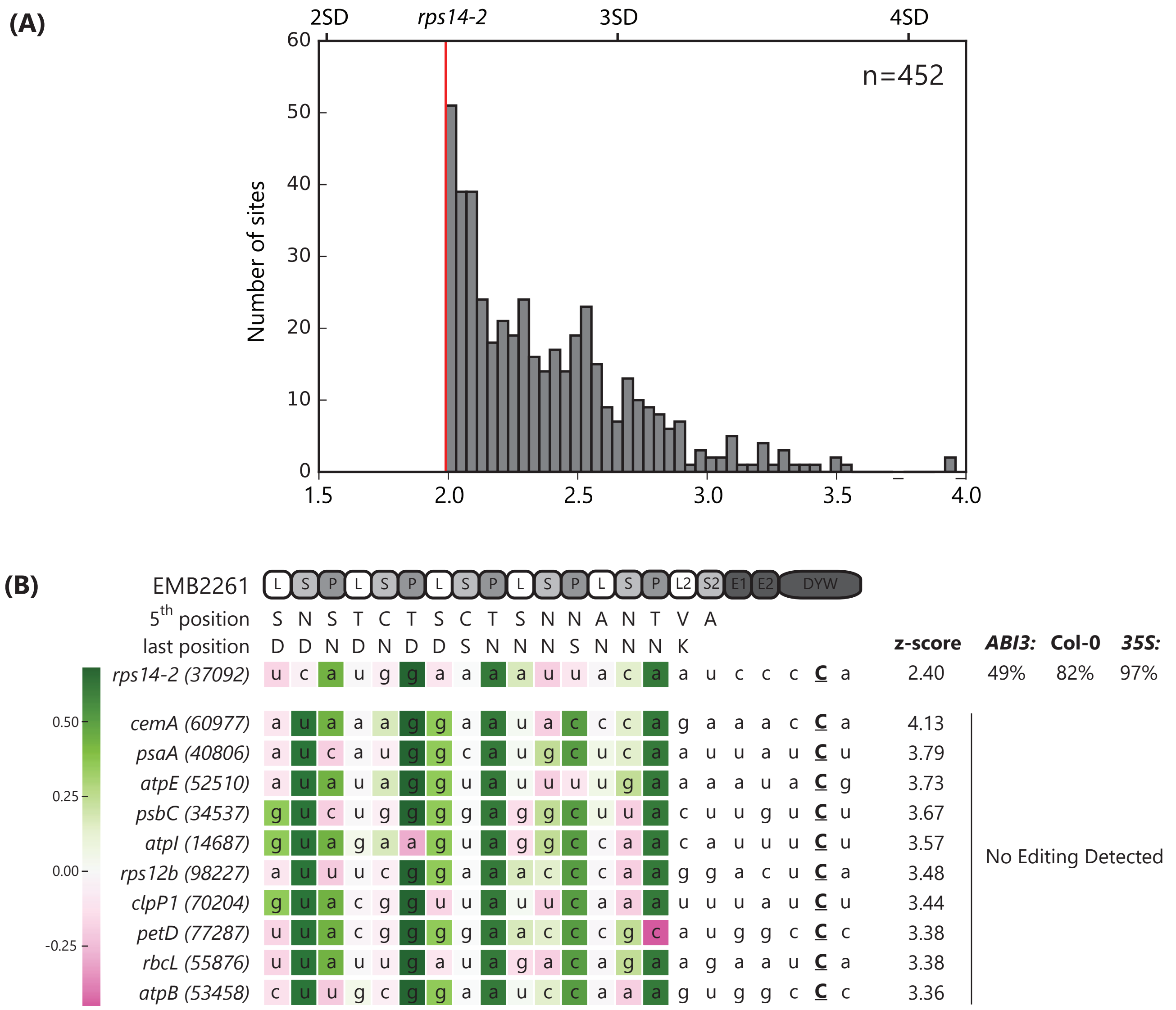

FIGURE S2. Candidates predicted to be edited by EMB2261.

(A) Distribution of prediction scores of potential EMB2261 editing sites across the entire *Arabidopsis* chloroplast genome (n=452) that is equal or higher than the score of *rps14-2* indicated by the red line. The mean score was calculated from scores of all potential YC editing sites predicted against EMB2261 (n=37,888). "2SD", "3SD" and "4SD" indicate two, three, and four standard deviation(s) from the mean. YC was specified since there is very rarely a G observed at the -1 position of any *Arabidopsis* chloroplast editing site. The only case reported so far is the *ndhG* editing site in *Arabidopsis* ecotype Cvi-0 (Tillich *et al.*, 2005).

(B) Alignments of EMB2261 motifs with the top 10 predicted editing sites within the coding sequences across the *Arabidopsis* chloroplast genome, in comparison with *rps14-2*. The nucleotides are coloured according to the alignment scores (Table S1).

(A)

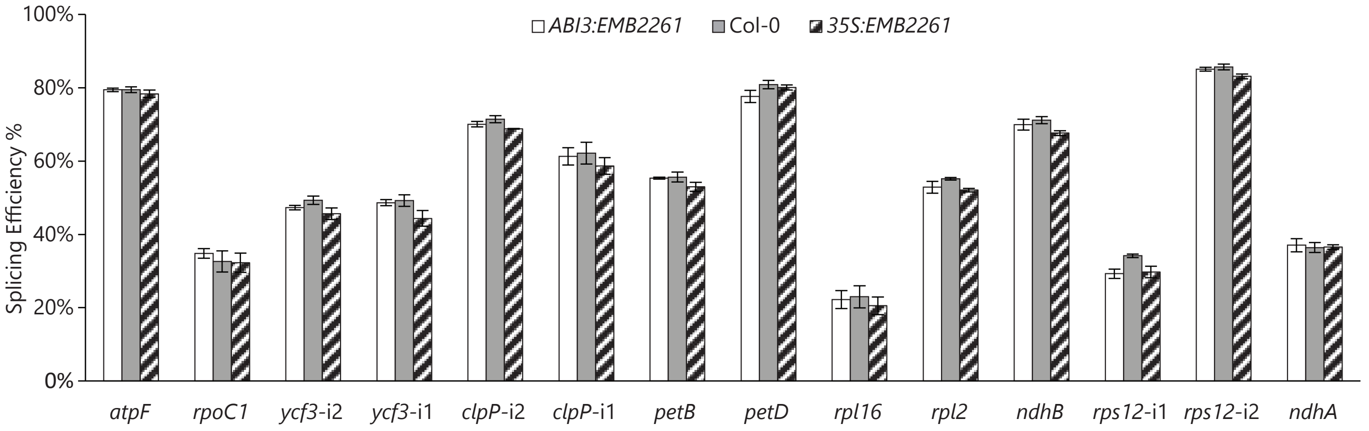

(B)

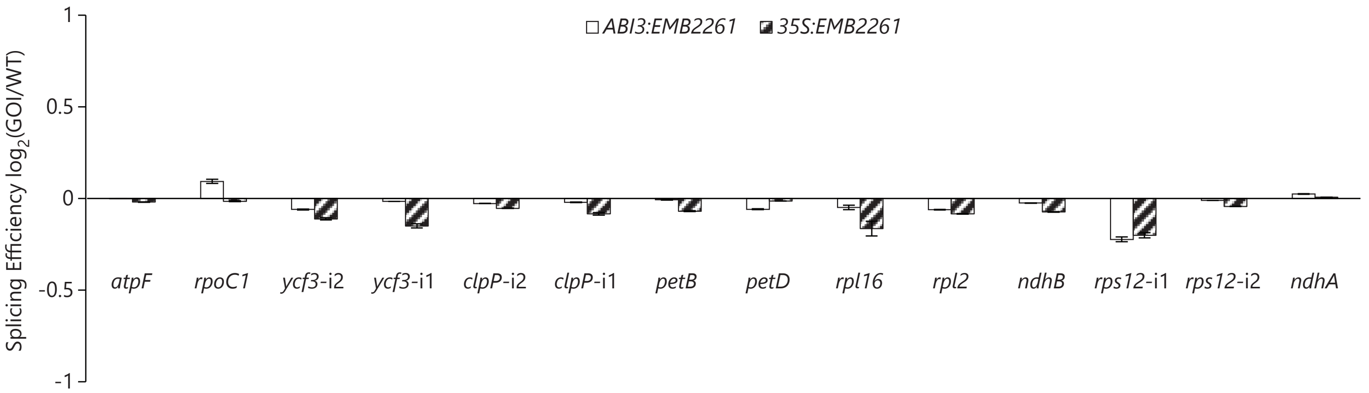

FIGURE S3. Splicing efficiency of chloroplast introns in *ABI3:EMB2261*, Col-0 and *35S:EMB2261* samples quantified based on the RNA-seq data.

(A) Splicing efficiency calculated by the chloroseq package (Castandet *et al.*, 2016). Error bars show SE, n=3.

(B) Splicing efficiency as log2 ratio of either *ABI3:EMB2261* or *35S:EMB2261* (GOIs) compared to Col-0 (WT). Error bars show SE, n=3.

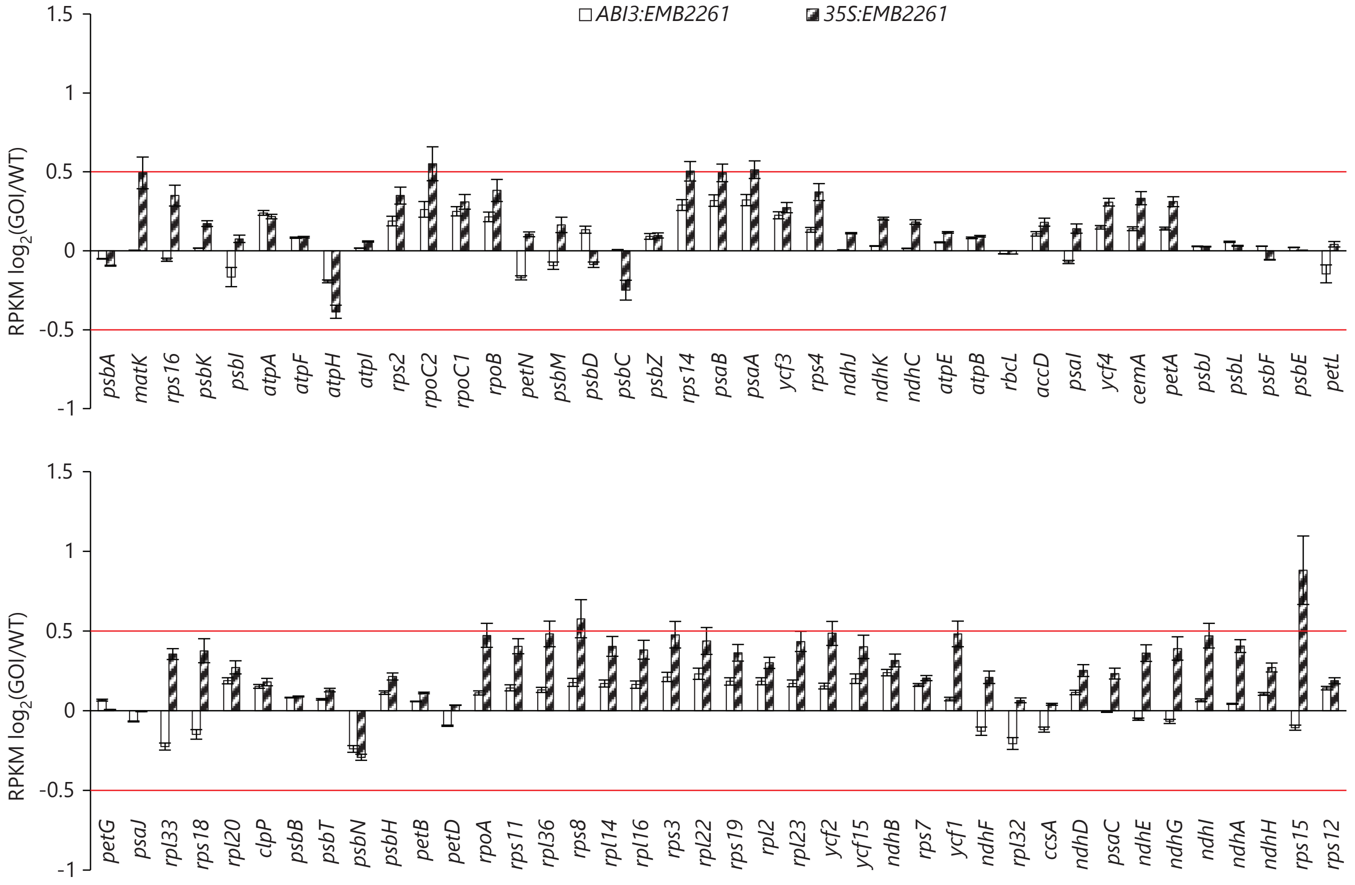

FIGURE S4. Chloroplast gene expression quantified as RPKM (Reads Per Kilobase of transcript per Million mapped reads) based on the RNA-seq data. The vertical axis indicate the log2 ratio of RPKM values of either *ABI3*:*EMB2261* or *35S*:*EMB2261* (GOIs) compared to Col-0 (WT). Error bars show SE, n=3.

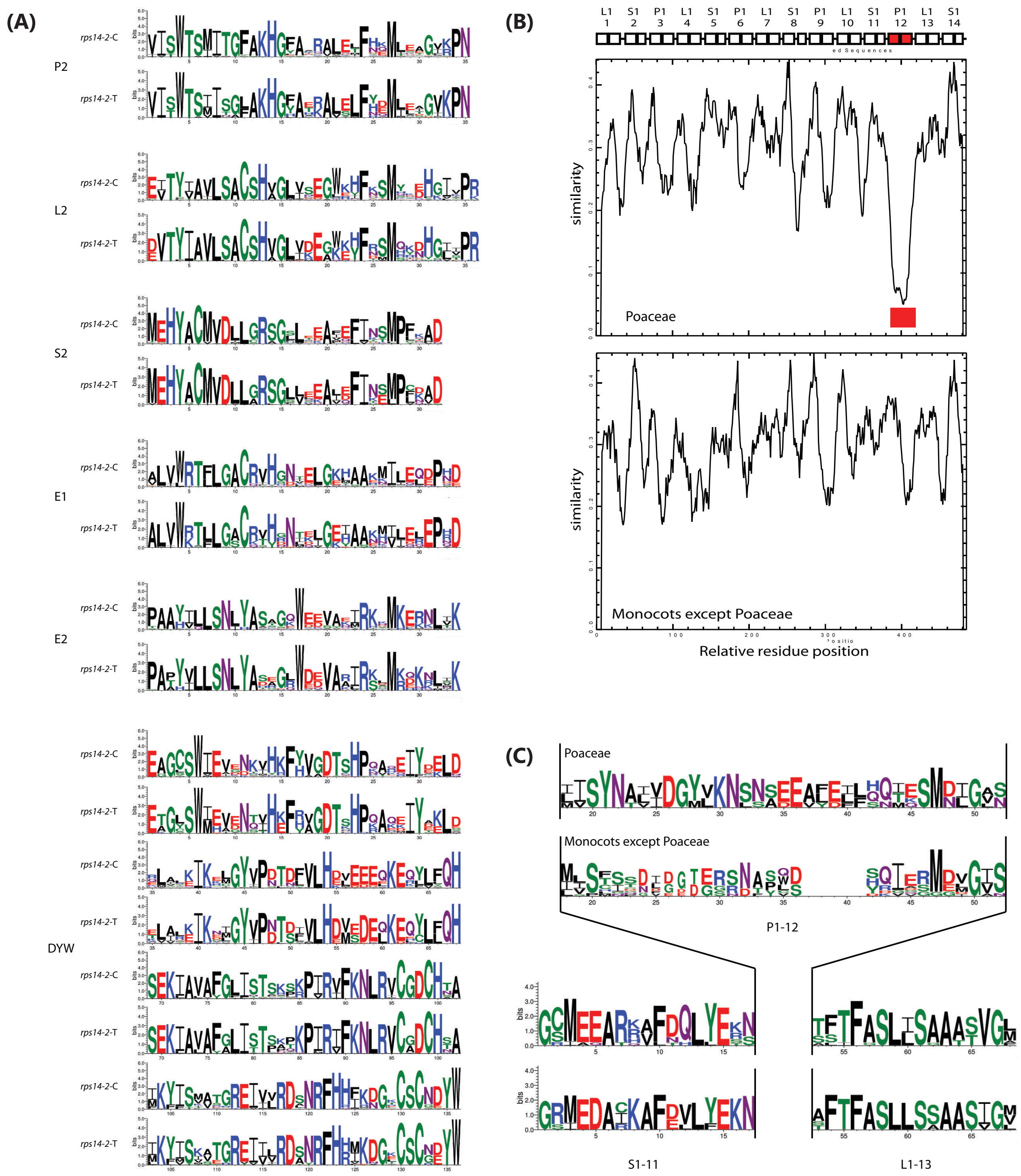

FIGURE S5. Sequence conservation and variation among EMB2261 orthologues.

(A) Consensus sequence logos for P2, L2, S2, E1, E2 and DYW showing the conservation of amino acids between the species with and without the need for *rps14-2* editing.

(B) Conservation of EMB2261 sequence between monocot species with and without the need for *rps14-2* editing. Top panel shows the similarity plot generated from 11 aligned sequences within the Poaceae family where *rps14-2* editing is no longer needed. Bottom panel shows the similarity plot generated from 5 aligned sequences from other monocot families where the *rps14-2* editing site is present. The red bar indicates the region of variation within the Poaceae family in contrast to other monocots, which corresponds to the 12th P1 motif.

(C) Consensus sequence logos of the 12th P1 motif and the upstream 11th S1 motif and downstream 13th L1 motif in Poaceae in comparison with other monocot species.

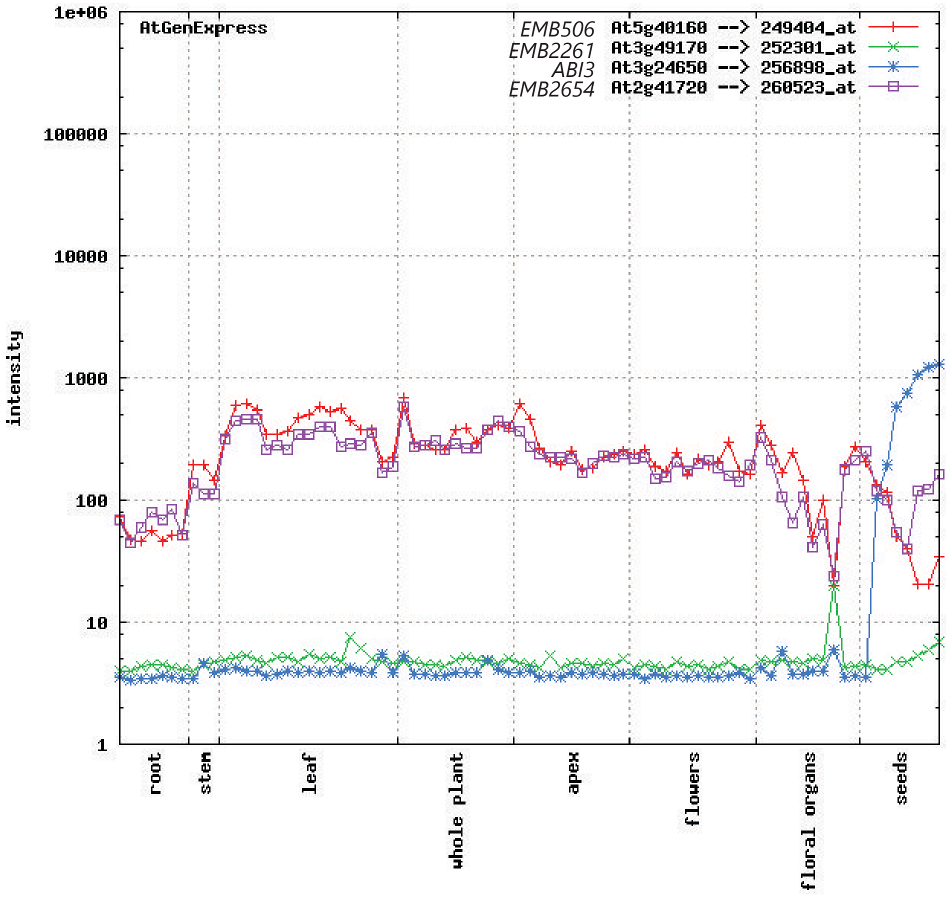

FIGURE S6. ***ABI3*** **vs.** ***EMB*** **gene expression profiles (Schmid** ***et al.*****, 2005).**
